# Knowledge, attitudes, and practices of patients with chronic kidney disease toward osteosarcopenia

**DOI:** 10.3389/fendo.2026.1739047

**Published:** 2026-03-12

**Authors:** Chunmei Yang, Wei Wei, Wenhui Chai, Tao Li, Yue Ma, Xuan Wang, Mei Liu, Bumaryam Abla, Hongtao Cai, Ping Li, Ying Liu, Jiao Liu

**Affiliations:** 1 Department of Geriatrics, People's Hospital of Changji Hui Autonomous Prefecture, Xinjiang, China; 2 Department of Infectious Diseases, People's Hospital of Changji Hui Autonomous Prefecture, Xinjiang, China; 3 Department of Science and Education, People's Hospital of Changji Hui Autonomous Prefecture, Xinjiang, China; 4 Department of Geriatrics, Changji Hui Autonomous Prefecture Hospital of Traditional Chinese Medicine, Xinjiang, China; 5 Department of Nutrition, People's Hospital of Changji Hui Autonomous Prefecture, Xinjiang, China

**Keywords:** attitude, chronic kidney disease, cross-sectional study, health knowledge, osteosarcopenia, practice

## Abstract

**Objective:**

To investigate the knowledge, attitudes, and practices of patients with chronic kidney disease regarding osteosarcopenia.

**Methods:**

This cross-sectional survey was conducted among patients with chronic kidney disease at People’s Hospital of Changji Hui Autonomous Prefecture, Xinjiang from February to September, 2025. Demographic information was obtained, and KAP scores were assessed using a questionnaire. The differences in KAP toward osteosarcopenia were compared among patients with different demographic characteristics. The factors influencing knowledge, attitudes, and practices were determined using logistic regression analyses. A path analysis was conducted to examine the relationships between the knowledge, attitudes, and practices dimensions.

**Results:**

This study enrolled 585 participants. The mean knowledge score was 6.33 ± 5.48 (on a maximum of 20, 31.65%), the mean attitude score was 34.33 ± 5.98 (on a maximum of 50, 68.66%), and the mean practice score was 27.89 ± 9.82 (on a maximum of 50, 55.78%). Being single (OR = 0.532, 95% CI: 0.322-0.876), requiring partial assistance with activities of daily living (OR = 0.247, 95% CI: 0.144-0.424), being bedridden (OR = 0.055, 95% CI: 0.012-0.246), score 9–12 for social support and environmental factors (OR = 1.674, 95% CI: 1.000-2.804), score 13–20 for social support and environmental factors (OR = 5.771, 95% CI: 2.971-11.209), score 5.0-6.9 for confidence level in preventing osteosarcopenia (OR = 3.933, 95% CI: 2.179-7.101), and score 7.0-10.0 for confidence level in preventing osteosarcopenia (OR = 4.227, 95% CI: 2.174-8.216) were independently associated with the knowledge scores. Knowledge had a direct and positive influence on attitude (β=0.682, P<0.001) and practice (β=0.523, P<0.001). Attitude positively directly influenced practice (β=0.344, P<0.001). Knowledge had a positive indirect influence on practice through attitude (β=0.235, P<0.001).

**Conclusion:**

Patients with chronic kidney disease had suboptimal knowledge, attitudes, and practices toward osteosarcopenia. Interventions should be designed to improve their knowledge, attitudes, and practices toward osteosarcopenia.

## Background

Chronic kidney disease (CKD) poses a rapidly escalating global health crisis, with a profound impact on morbidity, mortality, and healthcare systems. Its dramatically rising incidence and prevalence highlight its significance as a major public health concern (1–3). A multifaceted condition, CKD is defined by progressive loss of renal function and serious systemic complications, particularly musculoskeletal disorders (4, 5). It disrupts homeostasis, leading to complications from hypertension and hyperkalemia to cardiovascular disease and immune dysfunction (4, 5). The core musculoskeletal complication is CKD-mineral and bone disorder (CKD-MBD), resulting from disturbed mineral metabolism. This leads to a spectrum of bone diseases (renal osteodystrophy), elevating fracture risk and causing extraskeletal calcification, including life-threatening calciphylaxis (4, 5). Accompanying muscle pain, spasms, and weakness further diminish quality of life. These complexities intensify the clinical burden, complicating management and prognosis (4, 5).

Sarcopenia and osteoporosis are two highly prevalent and interrelated conditions in patients with chronic kidney disease (CKD), collectively referred to as “Sarcopenia-Osteoporosis Syndrome” or “Osteosarcopenia” (6–8). Sarcopenia is defined as a progressive and generalized loss of skeletal muscle mass, strength, and function, commonly associated with aging but highly aggravated by CKD due to metabolic disturbances, chronic inflammation, hormonal imbalances, and malnutrition (8, 9). Osteoporosis is characterized by reduced bone mineral density and impaired bone microarchitecture, leading to an increased fracture risk. In CKD, these bone changes are accelerated due to alterations in calcium, phosphate, and vitamin D metabolism, as well as secondary hyperparathyroidism (6, 7). The Osteosarcopenia highlights the deep biological and clinical crosstalk between bone and muscle deterioration in CKD; muscle and bone health are interdependent, and their decline is mutually reinforcing (6, 8). Diagnosis is complex due to overlapping symptoms, and management demands integrated strategies targeting both muscle and bone health (6, 8). Osteosarcopenia markedly elevates the risk of falls and fractures in CKD patients, with severe repercussions for patient outcomes and healthcare systems (6, 10).

Early recognition and management of osteosarcopenia in CKD are vital to reducing the high risk of fractures and physical decline in these patients (6, 8). While clinical diagnostics exist, successful intervention also depends on patients’ ability to identify and report symptoms, highlighting the importance of patient education (6). Consequently, self-management becomes a cornerstone in the treatment approach, empowering patients to maintain behaviors that support muscle and bone health—including physical activity, nutrition, fall prevention, and monitoring (6, 11, 12). This approach is linked to better clinical outcomes, fewer falls and fractures, improved quality of life, and lower healthcare costs. Ultimately, enhancing self-management first requires a clear understanding of patients’ current awareness and practices related to osteosarcopenia.

KAP (Knowledge, Attitude, and Practice) studies are foundational tools in public health for identifying gaps between what people know, believe, and do regarding a health issue (13, 14). The findings are critical for designing effective educational and interventional strategies (13, 14). However, despite the clinical focus on musculoskeletal health in CKD patients (15, 16), their personal KAP toward osteosarcopenia remains completely unexplored. Addressing this void is crucial, as unrecognized knowledge deficits, resistant attitudes, and inadequate self-management behaviors are likely key, modifiable barriers to improving patient outcomes and preventing fractures and functional decline.

Therefore, this study aimed to assess the KAP of patients with CKD toward osteosarcopenia. The findings will provide valuable data to inform the design of targeted health education programs, enhance patient self-management, and ultimately improve the quality of life and prognosis for patients with CKD.

## Methods

### Study design and participants

This cross-sectional survey was conducted among CKD patients at People’s Hospital of Changji Hui Autonomous Prefecture, Xinjiang from February to September, 2025. Inclusion criteria were: (1) had CKD (stages 1–5 per KDIGO criteria); (2) were ≥18 years old; (3) could comprehend and complete the e-questionnaire; (4) were clinically stable (no recent major surgery or acute events) for 3 months; and (5) provided informed consent. Exclusion criteria were: (1) major cognitive/psychiatric disorders; (2) inability to complete the Chinese questionnaire; (3) end-stage comorbidities (life expectancy <6 months); (4) major clinical events within 3 months; (5) pregnancy/lactation; (6) poor data quality per prespecified checks; or (7) declined data use. The study was approved by the Ethics Committee of People’s Hospital of Changji Hui Autonomous Prefecture, Xinjiang (Approval No.: SBGJ202412110005). The participants provided informed consent before completing the survey.

### Questionnaire introduction

The questionnaire was designed based on the recent relevant literature on osteosarcopenia (7, 11, 12). The questionnaire was revised to improve content validity based on feedback from two experts consulted via correspondence, who independently reviewed the relevance, clarity, and wording of each item, and provided suggestions for refinement of item expressions and structure. A pilot test was performed in 42 participants, revealing good internal consistency of the overall questionnaire (Cronbach’s α = 0.902), with subscale Cronbach’s α values of 0.919 for knowledge, 0.842 for attitude, and 0.672 for practice. During data processing, questionnaires with missing key items were excluded from the final analysis. For the knowledge dimension, responses of “not familiar” were treated as valid responses and scored as 0 points according to the predefined scoring scheme, rather than being considered missing values. Exploratory factor analysis was performed to examine construct validity, and the Kaiser–Meyer–Olkin (KMO) measure indicated good sampling adequacy (KMO > 0.80), supporting the suitability of the questionnaire structure for subsequent formal investigation. The participants were also requested to identify any statements that were unclear or difficult to understand to ensure face validity.

The final questionnaire, designed and administered in Chinese, consisted of four sections: demographic information and knowledge, attitude, and practice dimensions. The knowledge dimension included 11 items. Item K8 was a trap question for quality control (“The capital of China is Shanghai.”). The remaining 10 items were scored as follows: “very familiar” = 2 points, “heard of it” = 1 point, and “not familiar” = 0 points, with a total score ranging from 0 to 20. The attitude dimension consisted of 10 items, assessed using a 5-point Likert scale ranging from “strongly disagree” (1 point) to “strongly agree” (5 points), with a total score ranging from 10 to 50. The practice dimension consisted of 10 items, assessed using a 5-point Likert scale ranging from “always” (5 points) to “never” (1 point), with a total score ranging from 10 to 50.

The cross-sectional study was conducted using an online, self-administered questionnaire via the Wenjuanxing platform.

### Statistical analysis

The KAP scores in each dimension were tested for normality using the Kolmogorov-Smirnov test. Normally distributed data were expressed as means ± standard deviations and analyzed using Student’s t-test (comparison of two groups/levels) or ANOVA (comparison of three or more groups/levels). Non-normally distributed data were expressed as medians (25th percentile, 75th percentile) and analyzed using the Mann-Whitney U-test (for comparison of two groups/levels) or the Kruskal-Wallis H-test (for comparison of three or more groups/levels). Categorical variables were expressed as n (%)and analyzed using the chi-squared test or Fisher’s exact test. Correlations among dimensions were assessed using the Pearson correlation coefficient when the data followed a normal distribution, or the Spearman correlation coefficient when they did not. Each dimension score was treated as the dependent variable for univariate and multivariable logistic regression analyses to assess the relationship between demographic characteristics and dimension scores. The dependent variables were categorized using the median of each dimension score. Variables with P<0.05 in univariate analyses were included in the multivariable analysis. All analyses were performed using R version 4.3.2. P-values <0.05 were considered statistically significant, with values reported to three decimal places.

Based on the KAP theoretical framework, a structural equation model (SEM) was applied to test whether attitude mediated the pathway from knowledge to practice. Indirect and direct effects were calculated and compared using path analysis. The model fit was assessed using root mean square error of approximation (RMSEA) <0.08, standardized root mean square residual (SRMR) <0.08, Tucker-Lewis index (TLI) >0.8, and comparative fit index (CFI) >0.8. If these thresholds were not met, path analysis was used to test for mediation effects. SEM and path analyses were performed using Stata 18.0 (IBM, Armonk, NY, USA).

## Results

### Characteristics of the participants

A total of 610 participants were initially enrolled in this study. Following data quality checks, 25 questionnaires were excluded due to incorrect answers to a trap question (n=6), an excessively short response time of <90 seconds (n=17), or obvious logical errors in demographic responses (n=2). Consequently, 585 valid questionnaires were included in the final analysis. In the formal study, the internal consistency and construct validity of the overall scale and its subscales were satisfactory. The Cronbach’s α coefficient for the total scale was 0.9667, with coefficients of 0.9556, 0.9479, and 0.9389 for the knowledge, attitude, and practice subscales, respectively. The KMO value for the total scale was 0.9480.

Age was distributed as follows: 34.2% were under 69 years old, 30.6% were between 70 and 79 years old, and 35.2% were 80 years or older. Most were of Han ethnicity (77.9%). Regarding anthropometric measures, 45.1% had a height <160 cm, and 27.5% had a body weight >71 kg. The majority resided in urban areas (73.3%) and had a primary school education or higher (89.7%), with 19.3% holding an associate degree or higher. Occupationally, over half were unemployed or retired (56.2%), and most reported a monthly per capita income between 3000 and 5000 RMB (53.7%). Most participants were married (72.6%) and had been diagnosed with CKD for <1 year (41.5%). Diabetes was present in 31.1% of subjects. Regarding falls during the previous year, 77.4% reported none. More than half (55.7%) were on current medications; specifically, 46.7% used calcium supplements, 30.1% vitamin D, and 26.5% phosphate binders. Most participants had never smoked (69.9%) or consumed alcohol (73.5%). In terms of daily activities, 65.6% were fully independent, and for social support, most individuals had moderate to high scores (> 9 in 69.9%). Confidence in preventing osteosarcopenia was moderate to high (>5.0) in 76.6% of participants (**Table 1**).

**Table 1:** Characteristics of the participants.

Variables	n (%)	Knowledge	P	Attitude	P	Practice	P
		mean ± SD		mean ± SD		mean ± SD	
Total score	585 (100.0)	6.33 ± 5.48		34.33 ± 5.98		27.89 ± 9.82	
Gender			0.259		0.929		0.045
Male	249 (42.6)	6.63 ± 5.51		34.36 ± 5.65		28.84 ± 10.10	
Female	336 (57.4)	6.11 ± 5.45		34.31 ± 6.22		27.19 ± 9.57	
Age (years)			<0.001		<0.001		<0.001
<69	200 (34.2)	8.47 ± 5.96		36.65 ± 5.42		30.56 ± 9.88	
70-79	179 (30.6)	6.15 ± 4.87		34.02 ± 5.30		27.73 ± 9.99	
≥80	206 (35.2)	4.42 ± 4.72		32.35 ± 6.31		25.44 ± 8.97	
Ethnicity			0.016		0.011		0.036
Han	456 (77.9)	6.04 ± 5.07		34.00 ± 5.78		27.44 ± 9.56	
Other	129 (22.1)	7.36 ± 6.66		35.51 ± 6.53		29.49 ± 10.59	
Height (cm)			0.019		0.103		0.077
≤160	264 (45.1)	5.67 ± 5.24		33.88 ± 6.15		26.73 ± 9.22	
161-165	98 (16.8)	7.61 ± 6.17		35.30 ± 6.34		28.60 ± 10.84	
166-170	105 (17.9)	6.31 ± 5.14		33.84 ± 5.20		28.76 ± 10.10	
≥171	118 (20.2)	6.76 ± 5.53		34.98 ± 5.85		29.11 ± 9.83	
Weight (kg)			0.926		0.645		0.242
≤50	76 (13.0)	6.04 ± 6.04		33.55 ± 7.34		26.13 ± 9.69	
51-60	175 (29.9)	6.37 ± 5.65		34.35 ± 6.27		27.53 ± 9.96	
61-70	173 (29.6)	6.51 ± 5.48		34.61 ± 5.51		28.77 ± 10.05	
≥71	161 (27.5)	6.24 ± 5.04		34.39 ± 5.43		28.16 ± 9.44	
Place of residence			0.121		0.017		0.293
Rural/suburban	156 (26.7)	5.75 ± 5.72		33.35 ± 6.44		27.18 ± 9.92	
Urban	429 (73.3)	6.55 ± 5.38		34.69 ± 5.77		28.15 ± 9.79	
Education level			<0.001		<0.001		<0.001
Illiterate	60 (10.3)	5.50 ± 6.07		32.67 ± 7.08		24.93 ± 10.89	
Primary school	157 (26.8)	5.46 ± 5.26		33.02 ± 6.10		26.34 ± 9.57	
Junior high school	127 (21.7)	6.17 ± 5.26		33.97 ± 5.69		27.48 ± 9.01	
Senior high school/technical secondary school	128 (21.9)	6.23 ± 5.23		34.84 ± 5.58		28.93 ± 9.59	
Associate degree and above	113 (19.3)	8.29 ± 5.58		36.87 ± 5.08		30.88 ± 9.96	
Type of occupation			<0.001		<0.001		<0.001
Manual labor	97 (16.6)	5.96 ± 5.54		34.30 ± 5.74		28.03 ± 9.45	
Mental labor	108 (18.5)	10.19 ± 5.43		37.69 ± 5.77		35.94 ± 9.30	
Unemployed/retired	329 (56.2)	4.85 ± 4.35		33.06 ± 5.37		24.85 ± 8.20	
Other	51 (8.7)	8.45 ± 7.30		35.49 ± 7.59		30.18 ± 11.10	
Monthly income per capita (RMB)			<0.001		<0.001		<0.001
<3000	109 (18.6)	4.70 ± 5.23		32.03 ± 6.16		23.90 ± 9.21	
3000-5000	314 (53.7)	5.65 ± 5.06		34.21 ± 5.55		26.86 ± 9.26	
>5000	162 (27.7)	8.77 ± 5.66		36.12 ± 6.13		32.56 ± 9.58	
Marital status			<0.001		0.02		0.003
Married	425 (72.6)	6.84 ± 5.45		34.68 ± 5.81		28.64 ± 9.85	
single	160 (27.4)	4.99 ± 5.34		33.39 ± 6.34		25.89 ± 9.50	
Duration since diagnosis of chronic kidney disease			0.002		0.039		<0.001
<1 year	243 (41.5)	5.74 ± 4.83		33.59 ± 5.13		25.37 ± 8.72	
1–3 years	164 (28.0)	7.59 ± 6.07		34.95 ± 6.29		30.61 ± 9.91	
>3 years	178 (30.4)	5.99 ± 5.59		34.78 ± 6.65		28.81 ± 10.37	
With diabetes			0.131		0.134		0.865
Yes	182 (31.1)	5.82 ± 4.93		33.78 ± 5.95		27.79 ± 9.10	
No	403 (68.9)	6.56 ± 5.70		34.58 ± 5.99		27.94 ± 10.14	
Number of falls in the past year			0.001		0.084		0.032
0 times	453 (77.4)	6.70 ± 5.51		34.57 ± 5.75		27.96 ± 9.83	
1 time	104 (17.8)	5.57 ± 5.31		33.88 ± 6.71		28.81 ± 9.83	
≥2 times	28 (4.8)	3.18 ± 4.21		32.18 ± 6.45		23.36 ± 8.81	
Current medications (multiple choice)			<0.001		<0.001		<0.001
No	259 (44.3)	4.76 ± 4.47		32.66 ± 5.52		23.76 ± 8.14	
Yes	326 (55.7)	7.58 ± 5.88		35.66 ± 6.01		31.17 ± 9.82	
Calcium supplements	273 (46.7)	7.77 ± 5.93		35.85 ± 6.11		31.81 ± 9.91	
Vitamin D	176 (30.1)	8.65 ± 6.33		36.75 ± 6.10		32.81 ± 10.22	
Phosphate binders	155 (26.5)	6.74 ± 5.58		35.35 ± 6.48		30.70 ± 10.02	
Smoking history			0.342		0.579		0.028
Never smoked	409 (69.9)	6.14 ± 5.40		34.23 ± 6.05		27.19 ± 9.53	
Quit smoking	86 (14.7)	7.06 ± 5.74		34.95 ± 5.83		29.91 ± 9.62	
Currently smoking	90 (15.4)	6.53 ± 5.59		34.18 ± 5.85		29.13 ± 10.97	
Alcohol consumption history			0.001		0.023		<0.001
Never	430 (73.5)	6.02 ± 5.44		34.02 ± 5.97		27.02 ± 9.70	
Occasionally (<1 time/week)	74 (12.6)	7.81 ± 5.47		35.15 ± 5.19		29.14 ± 9.82	
Frequently (≥1 time/week)	26 (4.4)	9.27 ± 5.55		37.42 ± 5.20		35.54 ± 10.95	
Previously drank, now abstinent	55 (9.4)	5.42 ± 5.08		34.24 ± 6.95		29.36 ± 8.48	
Ability in daily activities			<0.001		<0.001		<0.001
Fully independent	384 (65.6)	7.99 ± 5.63		36.09 ± 5.58		30.42 ± 10.21	
Requires partial assistance	171 (29.2)	3.57 ± 3.48		31.32 ± 5.03		23.26 ± 6.71	
Bedridden	30 (5.1)	0.90 ± 1.79		29.00 ± 6.05		21.83 ± 7.25	
Social support and environmental factors			<0.001		<0.001		<0.001
4-8	176 (30.1)	3.01 ± 3.05		30.26 ± 5.05		19.61 ± 5.60	
9-12	216 (36.9)	5.46 ± 4.60		33.96 ± 4.94		25.83 ± 6.24	
13-20	193 (33.0)	10.34 ± 5.66		38.47 ± 5.06		37.74 ± 7.50	
Confidence level in preventing osteosarcopenia			<0.001		<0.001		<0.001
<5.0	137 (23.4)	2.93 ± 4.36		30.73 ± 6.12		21.65 ± 7.15	
5.0-6.9	246 (42.1)	6.22 ± 4.76		34.12 ± 5.18		27.30 ± 9.13	
7.0-10.0	202 (34.5)	8.78 ± 5.73		37.03 ± 5.44		32.84 ± 9.62	

### Knowledge, attitude, and practice

The mean knowledge score was 6.33 ± 5.48 (on a maximum of 20, 31.65%), the mean attitude score was 34.33 ± 5.98 (on a maximum of 50, 68.66%), and the mean practice score was 27.89 ± 9.82 (on a maximum of 50, 55.78%) (**Table 1**).

The knowledge item with the highest score was K5 (26.5% very familiar), while the item with the lowest score was K10 (5.47% very familiar) (**Table 2**). Most respondents either “strongly agreed” or “agreed” with statements emphasizing the importance of and willingness to prevent or manage osteosarcopenia (items A1 and A4-A10). Notably, 64.28% attached importance to osteosarcopenia, and 66.54% indicated willingness to take action to prevent or improve the condition. A majority (75.64%) agreed that consuming sufficient protein and calcium daily could prevent osteosarcopenia, and 81.95% expressed willingness to follow doctors’ advice regarding appropriate exercise. Conversely, fewer participants reported actively seeking information or treatment advances (32.31% “strongly agree” or “agree”), and 44.23% remained neutral or disagreed with active learning (item A2). Most participants recognized that osteosarcopenia could affect quality of life (84.96% “strongly agree”, “agree”, or “neutral”), and 88.09% viewed CKD as increasing the risk of developing osteosarcopenia (**Table 3**). Participants’ practices related to the prevention and management of osteosarcopenia showed considerable variability across behaviors. Routine biochemical monitoring was performed “always” or “often” by 28.72% of participants in the past six months, while 37.95% engaged in such monitoring “sometimes.” Adjusting protein intake as per medical advice was carried out “always” or “often” by 34.87%, but 27.86% reported “never” doing so. Daily recording of dietary calcium and protein intake was less common, with only 22.05% reporting this behavior “always” or “often,” while 64.28% reported doing so “rarely” or “never.” Exercise-related practices remained suboptimal: 25.64% reported “always” or “often” engaging in resistance training, and 24.96% practiced balance training with a similar frequency; yet, a substantial proportion rarely or never engaged in these activities (59.83% for resistance training, 61.37% for balance training). Achieving a daily walking exercise threshold of >6000 steps was reported “always” or “often” by 35.05%. Adherence to prescribed calcium/vitamin D supplementation was comparatively higher, with 41.54% “always” or “often” taking medications as recommended. Regular dual-energy X-ray absorptiometry (DXA) bone mineral density testing was performed “always” or “often” by 41.88% of participants, while 39.31% reported such testing “rarely” or “never.” Home modifications to prevent falls were implemented “always” or “often” by 28.37%, while 43.25% undertook these measures “rarely” or “never.” Avoiding lifting objects heavier than 5 kg was among the best-adhered-to practices, with 58.12% reporting that they “always” or “often” engaged in this preventive measure (**Table 4**).

**Table 2:** Knowledge dimension distribution.

Knowledge	Very familiar	Heard of it	Not sure
1.Osteosarcopenia is a geriatric condition in which sarcopenia and osteoporosis coexist.	44 (7.52%)	182 (31.11%)	359 (61.37%)
2.Aging, nutritional deficiency, hormonal changes, and lack of exercise all play important roles in the development and progression of osteosarcopenia.	41 (7.01%)	195 (33.33%)	349 (59.66%)
3.Lack of exercise is an important risk factor. Long-term inactivity can lead to muscle atrophy, decreased muscle strength, and reduced endurance.	89 (15.21%)	158 (27.01%)	338 (57.78%)
4.Patients with osteosarcopenia may experience reduced muscle mass accompanied by vertebral compression fractures.	49 (8.38%)	352 (60.17%)	184 (31.45%)
5.Osteosarcopenia can be diagnosed through muscle assessment and bone assessment methods.	155 (26.5%)	272 (46.5%)	158 (27.01%)
6.Nutritional interventions for osteosarcopenia include ensuring sufficient protein intake by consuming foods such as lean meat, fish, eggs, and legumes, as well as supplementing with calcium and vitamin D.	53 (9.06%)	176 (30.09%)	356 (60.85%)
7.Aerobic exercises such as walking, jogging, and swimming, combined with strength training such as using dumbbells and resistance bands, can help increase muscle mass and strength and improve bone density.	50 (8.55%)	186 (31.79%)	349 (59.66%)
8.There is a close relationship between chronic kidney disease (CKD) and osteosarcopenia. They interact with each other, forming a vicious cycle that severely affects patients’ quality of life and prognosis.	75 (12.82%)	365 (62.39%)	145 (24.79%)
9.Patients with CKD are prone to osteosarcopenia mainly due to disorders of calcium-phosphorus metabolism, inadequate protein intake, and lack of exercise.	127 (21.71%)	222 (37.95%)	236 (40.34%)
10.CKD impairs kidney function, leading to reduced production of active vitamin D. Vitamin D is not only crucial for calcium absorption and bone health but also plays a role in muscle function.	32 (5.47%)	167 (28.55%)	386 (65.98%)

**Table 3:** Attitude dimension distribution.

Attitude	Strongly agree	Agree	Neutral	Disagree	Strongly disagree
1.You attach great importance to osteosarcopenia (P).	63 (10.77%)	152 (25.98%)	204 (34.87%)	71 (12.14%)	95 (16.24%)
2.You actively seek to learn about the latest knowledge or treatment advances regarding osteosarcopenia (P).	60 (10.26%)	129 (22.05%)	128 (21.88%)	156 (26.67%)	112 (19.15%)
3.You believe osteosarcopenia can seriously affect your quality of life (N).	72 (12.31%)	195 (33.33%)	230 (39.32%)	61 (10.43%)	27 (4.62%)
4.You are willing to take action to prevent or improve osteosarcopenia (P).	102 (17.44%)	274 (46.84%)	172 (29.4%)	27 (4.62%)	10 (1.71%)
5.You believe it is important for CKD patients to understand osteosarcopenia (P).	72 (12.31%)	201 (34.36%)	246 (42.05%)	58 (9.91%)	8 (1.37%)
6.You believe CKD increases the risk of developing osteosarcopenia (P).	60 (10.26%)	177 (30.26%)	259 (44.27%)	79 (13.5%)	10 (1.71%)
7.You believe that consuming sufficient protein and calcium daily can prevent osteosarcopenia (P).	138 (23.59%)	322 (55.04%)	106 (18.12%)	16 (2.74%)	3 (0.51%)
8.You feel it is very necessary to conduct screening for osteosarcopenia among CKD patients (P).	102 (17.44%)	207 (35.38%)	238 (40.68%)	34 (5.81%)	4 (0.68%)
9.You are willing to follow your doctor’s advice and engage in appropriate exercise to improve osteosarcopenia (P).	154 (26.32%)	271 (46.32%)	142 (24.27%)	15 (2.56%)	3 (0.51%)
10.You are willing to participate in health education activities aimed at preventing osteosarcopenia in CKD patients (P).	130 (22.22%)	247 (42.22%)	172 (29.4%)	34 (5.81%)	2 (0.34%)

**Table 4:** Practice dimension distribution.

Practice	Always	Often	Sometimes	Rarely	Never
1. Frequency of biochemical monitoring (serum calcium/phosphorus/vitamin D)	60 (10.26%)	108 (18.46%)	222 (37.95%)	163 (27.86%)	32 (5.47%)
2. Adjusting protein intake according to medical advice (e.g., high-quality low-protein diet).	82 (14.02%)	122 (20.85%)	116 (19.83%)	102 (17.44%)	163 (27.86%)
3. Actively recording daily dietary intake of calcium and protein.	51 (8.72%)	78 (13.33%)	80 (13.68%)	114 (19.49%)	262 (44.79%)
4. Resistance training (e.g., resistance bands/machine training).	61 (10.43%)	89 (15.21%)	85 (14.53%)	127 (21.71%)	223 (38.12%)
5. Balance training (e.g., single-leg standing, Tai Chi) to prevent falls.	61 (10.43%)	85 (14.53%)	80 (13.68%)	80 (13.68%)	279 (47.69%)
6. Walking exercise reaching ≥6,000 steps daily.	91 (15.56%)	114 (19.49%)	119 (20.34%)	136 (23.25%)	125 (21.37%)
7. Taking calcium/vitamin D supplements as prescribed.	81 (13.85%)	162 (27.69%)	195 (33.33%)	91 (15.56%)	56 (9.57%)
8. Regular dual-energy X-ray absorptiometry (DXA) bone mineral density testing.	42 (7.18%)	203 (34.7%)	110 (18.8%)	161 (27.52%)	69 (11.79%)
9. Home modifications for fall prevention (e.g., installing handrails, anti-slip mats).	69 (11.79%)	97 (16.58%)	166 (28.38%)	64 (10.94%)	189 (32.31%)
10. Avoiding lifting objects >5 kg.	168 (28.72%)	172 (29.4%)	142 (24.27%)	43 (7.35%)	60 (10.26%)

### Correlation analysis

Correlation analyses revealed significant positive correlations between knowledge and attitude scores (r = 0.716, P < 0.001), knowledge and practice scores (r = 0.655, P < 0.001), as well as between attitude and practice scores (r = 0.673, P < 0.001) (**Table 5**).

**Table 5:** Correlation analysis.

Spearman	Knowledge	Attitude	Practice
Knowledge	1.000		
Attitude	0.716 (P<0.001)	1.000	
Practice	0.655 (P<0.001)	0.673 (P<0.001)	1.000

### Multivariable analyses

Being single (OR = 0.532, 95% CI: 0.322-0.876, P = 0.013), requiring partial assistance with activities of daily living (OR = 0.247, 95% CI: 0.144-0.424, P<0.001), being bedridden (OR = 0.055, 95% CI: 0.012-0.246, P<0.001), score 9–12 for social support and environmental factors (OR = 1.674, 95% CI: 1.000-2.804, P = 0.050), score 13–20 for social support and environmental factors (OR = 5.771, 95% CI: 2.971-11.209, P<0.001), score 5.0-6.9 for confidence level in preventing osteosarcopenia (OR = 3.933, 95% CI: 2.179-7.101, P<0.001), and score 7.0-10.0 for confidence level in preventing osteosarcopenia (OR = 4.227, 95% CI: 2.174-8.216, P<0.001) were independently associated with the knowledge scores (**Table 6**).

**Table 6:** Univariate and multivariable analysis of the factors associated with the knowledge dimension scores.

Knowledge (>4 vs. <3)	Univariate analysis	P	Multivariable analysis	P
	OR (95% CI)		OR (95% CI)	
Gender				
Male				
Female	0.763 (0.544,1.066)	0.114		
Age (years)				
≤69				
70-79	0.574 (0.369,0.886)	0.013	1.469 (0.819,2.636)	0.197
≥80	0.262 (0.171,0.397)	<0.001	1.215 (0.636,2.320)	0.555
Ethnicity				
Han				
Other	1.020 (0.687,1.525)	0.922		
Height (cm)				
≤160				
161-165	1.500 (0.934,2.439)	0.097		
166-170	1.250 (0.792,1.988)	0.341		
≥171	1.565 (1.002,2.468)	0.051		
Weight (kg)				
≤50				
51-60	1.318 (0.766,2.269)	0.318		
61-70	1.357 (0.787,2.338)	0.272		
≥71	1.437 (0.827,2.496)	0.197		
Place of residence				
Rural/suburban				
Urban	1.566 (1.082,2.267)	0.017	1.113 (0.637,1.943)	0.707
Education level				
Illiterate				
Primary school	1.158 (0.638,2.109)	0.631	0.776 (0.342,1.759)	0.543
Junior high school	1.496 (0.808,2.785)	0.201	0.564 (0.232,1.371)	0.206
Senior high school/technical secondary school	1.843 (0.993,3.443)	0.053	0.616 (0.248,1.529)	0.296
Associate degree and above	4.238 (2.168,8.456)	<0.001	0.919 (0.343,2.461)	0.867
Type of occupation				
Manual labor				
Mental labor	3.889 (2.084,7.480)	<0.001	1.817 (0.766,4.307)	0.175
Unemployed/retired	0.943 (0.597,1.485)	0.802	1.642 (0.821,3.284)	0.161
Other	1.186 (0.599,2.373)	0.626	1.457 (0.527,4.024)	0.468
Monthly income per capita (RMB)				
<3000				
3000-5000	1.467 (0.948,2.277)	0.086	0.817 (0.431,1.546)	0.534
>5000	3.722 (2.222,6.313)	<0.001	1.471 (0.668,3.239)	0.338
Marital status				
Married				
single	0.401 (0.276,0.580)	<0.001	0.532 (0.322,0.876)	0.013
Duration since diagnosis of chronic kidney disease				
<1 year				
1–3 years	1.056 (0.703,1.590)	0.793		
>3 years	0.769 (0.519,1.137)	0.188		
With diabetes				
Yes				
No	1.216 (0.852,1.733)	0.279		
Number of falls in the past year				
0 times				
1 time	0.681 (0.443,1.047)	0.079	0.827 (0.431,1.584)	0.566
≥2 times	0.337 (0.147,0.733)	0.007	2.116 (0.636,7.042)	0.222
Current medications (multiple choice)				
Yes				
No	0.492 (0.351,0.687)	<0.001	0.624 (0.381,1.023)	0.061
Smoking history				
Never smoked				
Quit smoking	1.519 (0.936,2.512)	0.096		
Currently smoking	1.050 (0.663,1.678)	0.836		
Alcohol consumption history				
Never				
Occasionally (<1 time/week)	2.897 (1.650,5.358)	<0.001	2.017 (0.981,4.146)	0.056
Frequently (≥1 time/week)	2.664 (1.110,7.403)	0.039	0.991 (0.308,3.184)	0.987
Previously drank, now abstinent	0.959 (0.546,1.696)	0.884	0.922 (0.425,2.001)	0.837
Ability in daily activities				
Fully independent				
Requires partial assistance	0.190 (0.129,0.279)	<0.001	0.247 (0.144,0.424)	<0.001
Bedridden	0.039 (0.009,0.114)	<0.001	0.055 (0.012,0.246)	<0.001
Social support and environmental factors				
4-8				
9-12	2.509 (1.668,3.803)	<0.001	1.674 (1.000,2.804)	0.050
13-20	11.393 (6.946,19.211)	<0.001	5.771 (2.971,11.209)	<0.001
Confidence level in preventing osteosarcopenia				
<5.0				
5.0-6.9	4.795 (3.045,7.690)	<0.001	3.933 (2.179,7.101)	<0.001
7.0-10.0	10.776 (6.536,18.183)	<0.001	4.227 (2.174,8.216)	<0.001

The knowledge scores (OR = 1.324, 95% CI: 1.231-1.425, P<0.001), associate degree education or above (OR = 1.961, 95% CI: 1.568-15.697, P = 0.006), diagnosis of CKD for >3 years (OR = 2.473, 95% CI: 1.233-4.958, P = 0.011), frequent alcohol consumption (OR = 0.234, 95% CI: 0.062-0.892, P = 0.033), score 9–12 for social support and environmental factors (OR = 1.929, 95% CI: 1.068-3.485, P = 0.029), and score 13–20 for social support and environmental factors (OR = 3.991, 95% CI: 1.945-8.189, P<0.001) were independently associated with the attitude scores (**Table 7**).

**Table 7:** Univariate and multivariable analysis of the factors associated with the attitude dimension scores.

Attitude (>34 vs. <33)	Univariate analysis	P	Multivariable analysis	P
	OR (95% CI)		OR (95% CI)	
Knowledge	1.428 (1.345,1.515)	<0.001	1.324 (1.231,1.425)	<0.001
Gender				
Male				
Female	0.972 (0.700,1.349)	0.863		
Age (years)				
** ≤**69				
70-79	0.416 (0.272,0.630)	<0.001	0.771 (0.405,1.466)	0.427
** ≥**80	0.280 (0.184,0.420)	<0.001	0.859 (0.411,1.792)	0.684
Ethnicity				
Han				
Other	1.507 (1.016,2.250)	0.043	1.204 (0.659,2.201)	0.546
Height (cm)				
≤160				
161-165	1.165 (0.733,1.858)	0.519	0.589 (0.283,1.225)	0.157
166-170	0.937 (0.595,1.474)	0.778	0.605 (0.303,1.209)	0.155
≥171	1.557 (1.005,2.429)	0.049	1.227 (0.611,2.463)	0.565
Weight (kg)				
≤50				
51-60	1.338 (0.780,2.307)	0.291		
61-70	1.505 (0.876,2.599)	0.140		
≥71	1.315 (0.761,2.282)	0.328		
Place of residence				
Rural/suburban				
Urban	1.269 (0.879,1.834)	0.204		
Education level				
Illiterate				
Primary school	1.347 (0.732,2.529)	0.345	1.820 (0.685,4.834)	0.230
Junior high school	1.828 (0.977,3.490)	0.062	1.675 (0.586,4.786)	0.336
Senior high school/Technical secondary school	2.241 (1.198,4.281)	0.013	2.219 (0.761,6.469)	0.144
Associate degree and above	5.138 (2.648,10.254)	<0.001	4.961 (1.568,15.697)	0.006
Type of occupation				
Manual labor				
Mental labor	5.147 (2.669,10.388)	<0.001	1.346 (0.491,3.685)	0.564
Unemployed/Retired	0.522 (0.329,0.823)	0.005	0.554 (0.272,1.128)	0.104
Other	1.186 (0.599,2.373)	0.626	0.448 (0.151,1.331)	0.148
Monthly income per capita (RMB)				
<3000				
3000-5000	1.959 (1.246,3.126)	0.004	1.916 (0.920,3.990)	0.082
>5000	5.497 (3.266,9.426)	<0.001	1.756 (0.742,4.153)	0.200
Marital status				
Married				
single	0.737 (0.511,1.060)	0.100		
Duration since diagnosis of chronic kidney disease				
<1 year				
1–3 years	2.393 (1.599,3.606)	<0.001	1.771 (0.934,3.358)	0.080
>3 years	1.952 (1.321,2.896)	0.001	2.473 (1.233,4.958)	0.011
With diabetes				
Yes				
No	1.110 (0.782,1.576)	0.559		
Number of falls in the past year				
0 times				
1 time	1.041 (0.680,1.599)	0.853		
≥2 times	0.804 (0.369,1.731)	0.576		
Current medications (multiple choice)				
Yes				
No	0.310 (0.220,0.434)	<0.001	0.723 (0.427,1.225)	0.228
Smoking history				
Never smoked				
Quit smoking	1.464 (0.917,2.362)	0.113		
Currently smoking	1.149 (0.727,1.819)	0.553		
Alcohol consumption history				
Never				
Occasionally (<1 time/week)	1.626 (0.988,2.713)	0.059	0.698 (0.326,1.493)	0.354
Frequently (≥1 time/week)	2.357 (1.035,5.851)	0.049	0.234 (0.062,0.892)	0.033
Previously drank, now abstinent	1.257 (0.717,2.223)	0.426	1.063 (0.474,2.384)	0.882
Ability in daily activities				
Fully independent				
Requires partial assistance	0.179 (0.119,0.267)	<0.001	0.566 (0.308,1.043)	0.068
Bedridden	0.129 (0.047,0.305)	<0.001	0.570 (0.176,1.851)	0.349
Social support and environmental factors				
4-8				
9-12	3.361 (2.158,5.321)	<0.001	1.929 (1.068,3.485)	0.029
13-20	21.245 (12.614,36.924)	<0.001	3.991 (1.945,8.189)	<0.001
Confidence level in preventing osteosarcopenia				
<5.0				
5.0-6.9	1.886 (1.223,2.937)	0.005	0.806 (0.403,1.613)	0.543
7.0-10.0	5.123 (3.224,8.258)	<0.001	1.150 (0.533,2.480)	0.722

The knowledge scores (OR = 1.150, 95% CI: 1.052-1.257, P = 0.002), the attitude scores (OR = 1.102, 95% CI: 1.029-1.180, P = 0.006), age >80 years (OR = 2.869, 95% CI: 1.213-6.785, P = 0.016), being unemployed or retired (OR = 0.336, 95% CI: 0.144-0.787, P = 0.012), diagnosis of CKD for 1–2 years (OR = 2.288, 95% CI: 1.119-4.676, P = 0.023), no current medication (OR = 0.374, 95% CI: 0.206-0.679, P = 0.001), score 9–12 for social support and environmental factors (OR = 7.824, 95% CI: 3.800-16.106, P<0.001), score 13–20 for social support and environmental factors (OR = 42.340, 95% CI: 16.345-109.675, P<0.001), score 5.0-6.9 for confidence level in preventing osteosarcopenia (OR = 2.370, 95% CI: 1.105-5.081, P = 0.027), and score 7.0-10.0 for confidence level in preventing osteosarcopenia (OR = 3.631, 95% CI: 1.536-8.578, P = 0.003) were independently associated with the practice scores (**Table 8**).

**Table 8:** Univariate and multivariable analysis of the factors associated with the practice dimension scores.

Practice (>25 vs. <24)	Univariate analysis	P	Multivariable analysis	P
	OR (95% CI)		OR (95% CI)	
Knowledge	1.369 (1.295,1.447)	<0.001	1.150 (1.052,1.257)	0.002
Attitude	1.317 (1.255,1.382)	<0.001	1.102 (1.029,1.180)	0.006
Gender				
Male				
Female	0.812 (0.583,1.129)	0.216		
Age (years)				
≤69				
70-79	0.583 (0.384,0.880)	0.010	1.935 (0.907,4.128)	0.088
≥80	0.460 (0.307,0.684)	<0.001	2.869 (1.213,6.785)	0.016
Ethnicity				
Han				
Other	1.235 (0.833,1.842)	0.296		
Height (cm)				
≤160				
161-165	1.191 (0.748,1.902)	0.463	0.869 (0.368,2.055)	0.750
166-170	1.244 (0.791,1.967)	0.346	1.249 (0.573,2.720)	0.576
≥171	1.632 (1.050,2.557)	0.031	1.551 (0.648,3.713)	0.324
Weight (kg)				
≤50				
51-60	1.091 (0.636,1.873)	0.752		
61-70	1.410 (0.821,2.429)	0.213		
≥71	1.557 (0.900,2.703)	0.114		
Place of residence				
Rural/suburban				
Urban	1.058 (0.732,1.528)	0.764		
Education level				
Illiterate				
Primary school	0.920 (0.506,1.676)	0.783	0.570 (0.192,1.696)	0.312
Junior high school	1.276 (0.690,2.371)	0.437	0.520 (0.162,1.673)	0.273
Senior high school/technical secondary school	1.726 (0.931,3.219)	0.084	0.812 (0.240,2.748)	0.738
Associate degree and above	2.656 (1.396,5.111)	0.003	1.087 (0.300,3.943)	0.899
Type of occupation				
Manual labor				
Mental labor	4.707 (2.370,9.864)	<0.001	1.256 (0.367,4.307)	0.717
Unemployed/Retired	0.454 (0.284,0.718)	0.001	0.336 (0.144,0.787)	0.012
Other	0.998 (0.500,2.015)	0.996	0.382 (0.112,1.311)	0.126
Monthly income per capita (RMB)				
<3000				
3000-5000	1.659 (1.066,2.606)	0.026	2.056 (0.863,4.894)	0.104
>5000	5.231 (3.106,8.962)	<0.001	2.652 (0.960,7.324)	0.060
Marital status				
Married				
single	0.712 (0.494,1.026)	0.068		
Duration since diagnosis of chronic kidney disease				
<1 year				
1–3 years	3.168 (2.096,4.839)	<0.001	2.288 (1.119,4.676)	0.023
>3 years	2.206 (1.490,3.284)	<0.001	1.240 (0.572,2.688)	0.586
With diabetes				
Yes				
No	0.820 (0.575,1.167)	0.271		
Number of falls in the past year				
0 times				
1 time	1.358 (0.882,2.113)	0.169		
≥2 times	0.637 (0.288,1.370)	0.251		
Current medications (multiple choice)				
Yes				
No	0.205 (0.144,0.291)	<0.001	0.374 (0.206,0.679)	0.001
Smoking history				
Never smoked				
Quit smoking	1.925 (1.188,3.182)	0.009	2.029 (0.729,5.643)	0.175
Currently smoking	1.215 (0.769,1.933)	0.406	0.977 (0.387,2.467)	0.961
Alcohol consumption history				
Never				
Occasionally (<1 time/week)	1.509 (0.917,2.518)	0.109	0.573 (0.228,1.443)	0.238
Frequently (≥1 time/week)	4.084 (1.630,12.410)	0.006	0.711 (0.129,3.920)	0.695
Previously drank, now abstinent	1.999 (1.118,3.694)	0.022	1.355 (0.435,4.223)	0.600
Ability in daily activities				
Fully independent				
Requires partial assistance	0.286 (0.195,0.416)	<0.001	0.971 (0.476,1.982)	0.936
Bedridden	0.265 (0.116,0.570)	0.001	1.545 (0.424,5.625)	0.510
Social support and environmental factors				
4-8				
9-12	10.525 (6.220,18.648)	<0.001	7.824 (3.800,16.106)	<0.001
13-20	124.636 (60.970,277.131)	<0.001	42.340 (16.345,109.675)	<0.001
Confidence level in preventing osteosarcopenia				
<5.0				
5.0-6.9	2.682 (1.725,4.228)	<0.001	2.370 (1.105,5.081)	0.027
7.0-10.0	8.287 (5.102,13.726)	<0.001	3.631 (1.536,8.578)	0.003

### SEM and path analyses

**Figure 1:**
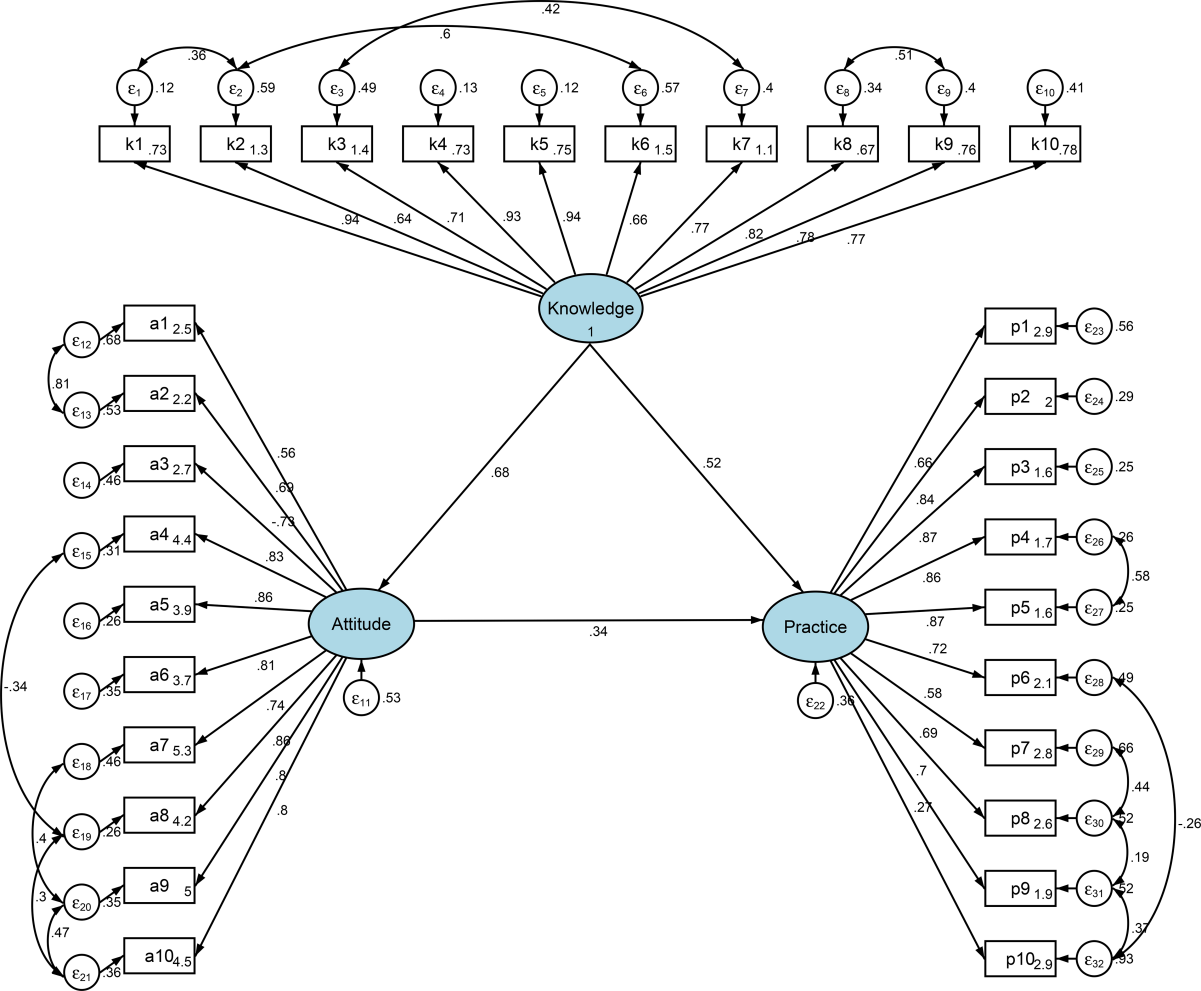
Structural equation modeling.

The fit index thresholds were met for TLI and CFI, but not for RMSEA and SRMR (**Table 9**). Therefore, although the SEM analysis results are provided (**Table 10**), the path analysis results were used to investigate how the KAP dimensions influenced each other. Knowledge had a direct and positive influence on attitude (β=0.682, P<0.001) and practice (β=0.523, P<0.001). Attitude positively directly influenced practice (β=0.344, P<0.001) (**Table 11**, **Figure 1**). Knowledge had a positive indirect influence on practice through attitude (β=0.235, P<0.001) (**Table 11**).

**Table 9:** Fit indexes of the SEM model.

Indicators	Reference	Results
RMSEA	<0.080	0.100
SRMR	<0.080	0.085
TLI	>0.800	0.856
CFI	>0.800	0.872

RMSEA, root mean square error of approximation; SRMR, standardized root mean square residual; TLI, Tucker-Lewis index; CFI, comparative fit index.

**Table 10:** SEM analysis.

Indicators		Estimate	P>|z|
Attitude			
	Knowledge	<0.001	27.58
Practice			
	Knowledge	<0.001	13.60
	Attitude	<0.001	37.60

**Table 11:** Path analysis.

Model paths		Total effects		Direct effect		Indirect effect	
		β (95% CI)	P	β (95% CI)	P	β (95% CI)	P
Attitude							
	Knowledge	0.682 (0.634, 0.731)	<0.001	0.682 (0.634, 0.731)	<0.001		
Practice							
	Knowledge	0.757 (0.718, 0.797)	<0.001	0.523 (0.447, 0.598)	<0.001	0.235 (0.178, 0.291)	<0.001
	Attitude	0.344 (0.265, 0.423)	<0.001	0.344 (0.265, 0.423)	<0.001		

## Discussion

This cross-sectional study evaluated the KAP concerning osteosarcopenia among patients with CKD. Given that both chronic kidney disease progression and osteosarcopenia are strongly age-related conditions, older adults represent a population at particularly high risk. Therefore, although the inclusion criterion allowed enrollment of adults aged ≥18 years, the predominance of participants aged over 69 years in this study reflects the real-world age distribution of CKD patients attending our center and the higher clinical burden of musculoskeletal complications in elderly patients. Our findings demonstrated that patients had suboptimal levels of KAP regarding this condition. Consequently, targeted interventions are warranted to improve their KAP, which may, in turn, enhance self-management and lead to better health outcomes.

The KAP scores of patients with CKD regarding osteosarcopenia (i.e., mean knowledge of 31.65%, attitude of 68.66%, and practice of 55.78%) indicate relatively low knowledge, moderate attitude, and suboptimal practice levels. No previous studies about the KAP toward osteosarcopenia in patients with CKD are available for direct comparisons, however, analogous data for osteoporosis in CKD populations offer relevant benchmarks. A recent cross-sectional study in China showed that CKD patients had a mean knowledge score of 60.6%, attitude score of 68%, and practice score of 78.4% toward osteoporosis (16). Two studies on the KAP of Chinese patients on hemodialysis toward sarcopenia revealed poor knowledge but more adequate attitude and practice (17, 18). Although the questionnaires differ, the comparative attitude scores were similar, suggesting CKD patients generally express moderately positive beliefs toward bone and muscle health. However, the knowledge and practice percentages in the present seem notably lower than those reported for osteoporosis KAP, especially for practice. It could be related to the study populations, but also to the nature of the investigated KAP (osteoporosis vs. osteosarcopenia) (16). Osteosarcopenia is recognized as a new clinical entity in CKD, but validated KAP tools and large-scale survey data are still emerging (6). Studies of osteoporosis in CKD almost universally report suboptimal knowledge and large practice gaps, though specific mean scores depend on the questionnaire and sample. Educational interventions and tailored programs are widely recommended to address these deficits (16, 19). Major gaps include a misunderstanding of calcium intake, medication risks, and underutilization of preventive strategies despite expressing concern or positive attitudes (16). Attitude scores consistently outpace actual knowledge and reported preventive practices among CKD cohorts internationally (16, 19). Hence, the KAP scores on osteosarcopenia in CKD patients appear lower for knowledge and practice compared to the existing literature on osteoporosis, though attitude levels remain consistent.

The present study reveals that knowledge regarding osteosarcopenia among chronic kidney disease patients remains conspicuously limited, with most respondents consistently reporting uncertainty across both foundational concepts and practical prevention strategies. Notably, fewer than 15% of participants considered themselves “very familiar” with items addressing the definition, risk factors, complications, or management of osteosarcopenia, and the majority (often exceeding 55%) selected “not sure,” indicating a significant gap in specific awareness and understanding. While diagnostic approaches achieved relatively high recognition, substantial ambiguity persisted regarding nutritional and exercise interventions, as well as the bidirectional relationship and risk mechanisms associated with CKD. This pattern of low knowledge and high uncertainty aligns with emerging literature, which highlights that musculoskeletal health risks are underemphasized in nephrology patient education and clinical practice. Collectively, these findings underscore an urgent need for targeted educational initiatives to promote recognition, early diagnosis, and management of osteosarcopenia in the CKD population, with future interventions ideally focusing on bridging gaps evident for both general and CKD-specific aspects of bone and muscle health (6, 9, 20). This discrepancy emphasizes the need for specific educational interventions for osteosarcopenia in the CKD population. Indeed, the path analysis revealed that knowledge has a positive influence on attitude and practice, and attitude has a positive influence on practice. Hence, improving knowledge should also lead to improved attitudes and practices. The interventions should be tailored to each patient and can take various forms, such as individualized teaching, family-based teaching, reading materials, interactive websites, and podcasts. Additional studies are necessary to design and test such interventions.

Multivariable analysis identified several independent factors significantly associated with knowledge scores regarding osteosarcopenia among CKD patients: being single, requiring partial assistance with activities of daily living, and being bedridden were all negative predictors, with lower odds ratios (OR = 0.532, 0.247, and 0.055, respectively), indicating that patients with greater dependency or without a partner were less likely to have higher knowledge. Conversely, greater social support and more favorable environmental factors were strongly positively associated with knowledge, with progressively higher odds ratios as scores increased (OR = 1.674 for scores 9–12 and OR = 5.771 for scores 13–20), as was greater confidence in preventing osteosarcopenia (OR = 3.933 for scores 5.0–6.9 and OR = 4.227 for scores 7.0-10.0). These findings underscore the influence of socio-environmental context and self-efficacy on patient understanding, while simultaneously highlighting that physical dependence and social isolation are barriers to effective health education in this population. The findings are consistent with the broader literature emphasizing the multifactorial nature of osteosarcopenia risk and knowledge gaps in CKD, which call for integrated support systems and targeted patient empowerment to bridge these deficits and improve clinical outcomes (6). These results could also suggest categories of patients who would need educational interventions more urgently.

This study had limitations. It was a single-center study conducted in Xinjiang that enrolled participants from one hospital and a single geographical region, which may limit the generalizability of the findings to other regions, ethnic populations, healthcare settings, and healthcare systems with different socioeconomic backgrounds, medical resources, and patient management patterns. In addition, key clinical variables such as CKD stage and dialysis status were not collected or included in the regression models. As these factors are strongly associated with disease severity, treatment burden, and patient education exposure, their omission may have resulted in residual confounding and should be considered when interpreting the associations between knowledge, attitudes, and practices. The cross-sectional nature of the study also prevents any analysis of causality. The questionnaire was designed to reflect the characteristics of the local study population; however, this approach may reduce comparability with other studies. Although a SEM analysis was performed to explore relationships among the KAP dimensions, it relies on statistical assumptions and predetermined hypotheses (21–23). Moreover, while the TLI and CFI values indicated acceptable model fit, the RMSEA and SRMR did not fully meet recommended thresholds, suggesting potential model misspecification. Therefore, the path analysis results should be interpreted with caution and primarily regarded as exploratory evidence of directional associations rather than definitive causal relationships. Finally, all KAP studies are susceptible to social desirability bias (24, 25).

In conclusion, patients with CKD had suboptimal KAP toward osteosarcopenia. Interventions should be designed to improve their KAP toward osteosarcopenia, which could translate into better self-management and patient outcomes.

## Data Availability

The original contributions presented in the study are included in the article/supplementary material. Further inquiries can be directed to the corresponding author.
